# Peering inside a cough or sneeze to explain enhanced airborne transmission under dry weather

**DOI:** 10.1038/s41598-021-89078-7

**Published:** 2021-05-10

**Authors:** Kai Liu, Majid Allahyari, Jorge S. Salinas, Nadim Zgheib, S. Balachandar

**Affiliations:** 1 Department of Mechanical and Aerospace Engineering, University of Florida, Gainesville, FL 32611 USA; 2 School of Engineering, Lebanese American University, Byblos, 1401 Lebanon

**Keywords:** Applied physics, Fluid dynamics

## Abstract

High-fidelity simulations of coughs and sneezes that serve as virtual experiments are presented, and they offer an unprecedented opportunity to peer into the chaotic evolution of the resulting airborne droplet clouds. While larger droplets quickly fall-out of the cloud, smaller droplets evaporate rapidly. The non-volatiles remain airborne as droplet nuclei for a long time to be transported over long distances. The substantial variation observed between the different realizations has important social distancing implications, since probabilistic outlier-events do occur and may need to be taken into account when assessing the risk of contagion. Contrary to common expectations, we observe dry ambient conditions to increase by more than four times the number of airborne potentially virus-laden nuclei, as a result of reduced droplet fall-out through rapid evaporation. The simulation results are used to validate and calibrate a comprehensive multiphase theory, which is then used to predict the spread of airborne nuclei under a wide variety of ambient conditions.

## Introduction

The COVID-19 disease, caused by the SARS-CoV-2 virus, has been detrimental to billions around the globe. One of the reasons this virus has turned into a global pandemic is its high infection rate^1,2^. The virus may be transmitted through direct contact with infected surfaces or through the airborne route^3,4^, i.e. by inhaling virus-laden droplets or aerosols ejected from an infected person through an expiratory event such as coughing, sneezing, or talking. While the importance of airborne route was initially being debated, it is now recognized as the dominant route for the spread of the SARS-CoV-2 virus^3,5,6^. To reduce the spread of the virus, social distancing guidelines have been put in place that require people to remain physically distant from one another by a distance of around two meters. The aforementioned guideline does not explicitly consider various parameters such as ambient conditions (temperature, humidity, wind speed, wind direction), the nature of the expiratory event (sneezing, coughing, breathing, singing, talking), or whether people are indoors or outdoors. All of which have been shown to be important factors in determining the risk of contagion^7–10^. This article focuses on the airborne transmission route, particularly through coughing and sneezing under dry and humid conditions.

No two coughs or sneezes are alike. For example, a violent sneeze of a large person generates a large puff containing a sizable number of potentially virus-laden droplets that extend farther than that of a child. Moreover, two nearly identical ejections may also show substantial differences as a result of their turbulent nature. Infinitesimal differences in the initial exhalation process can dramatically amplify and send a cough or sneeze careening in different paths, the so-called *butterfly effect*^11,12^. Such chaotic evolution must be properly accounted for in social distancing guidelines to safeguard against, not only average conditions, but also, extreme departures from the average. Nevertheless, there are important underlying universal properties that are common across all expiratory events^13^.

One of the objectives of the present work is to evaluate the accuracy of a recently proposed theoretical framework^14^ in predicting airborne transmission under a wide range of conditions. This work will demonstrate that quantities of interest to viral contagion, namely, the total volume, size distribution, and location of airborne droplet nuclei, can be well predicted with the theoretical framework. Large eddy simulations are used to support the theory by identifying and improving its approximations and obtaining the empirical coefficients needed in the theory. Once validated by the companion simulations, the advantage of the theory is that it can be readily used to investigate airborne contagion under a variety of ambient conditions. Such an approach is used to highlight an important effect of droplet evaporation - humid conditions lead to a substantially smaller volume of potentially virus-laden, airborne nuclei due to enhanced droplet settling.

**Figure 1:**
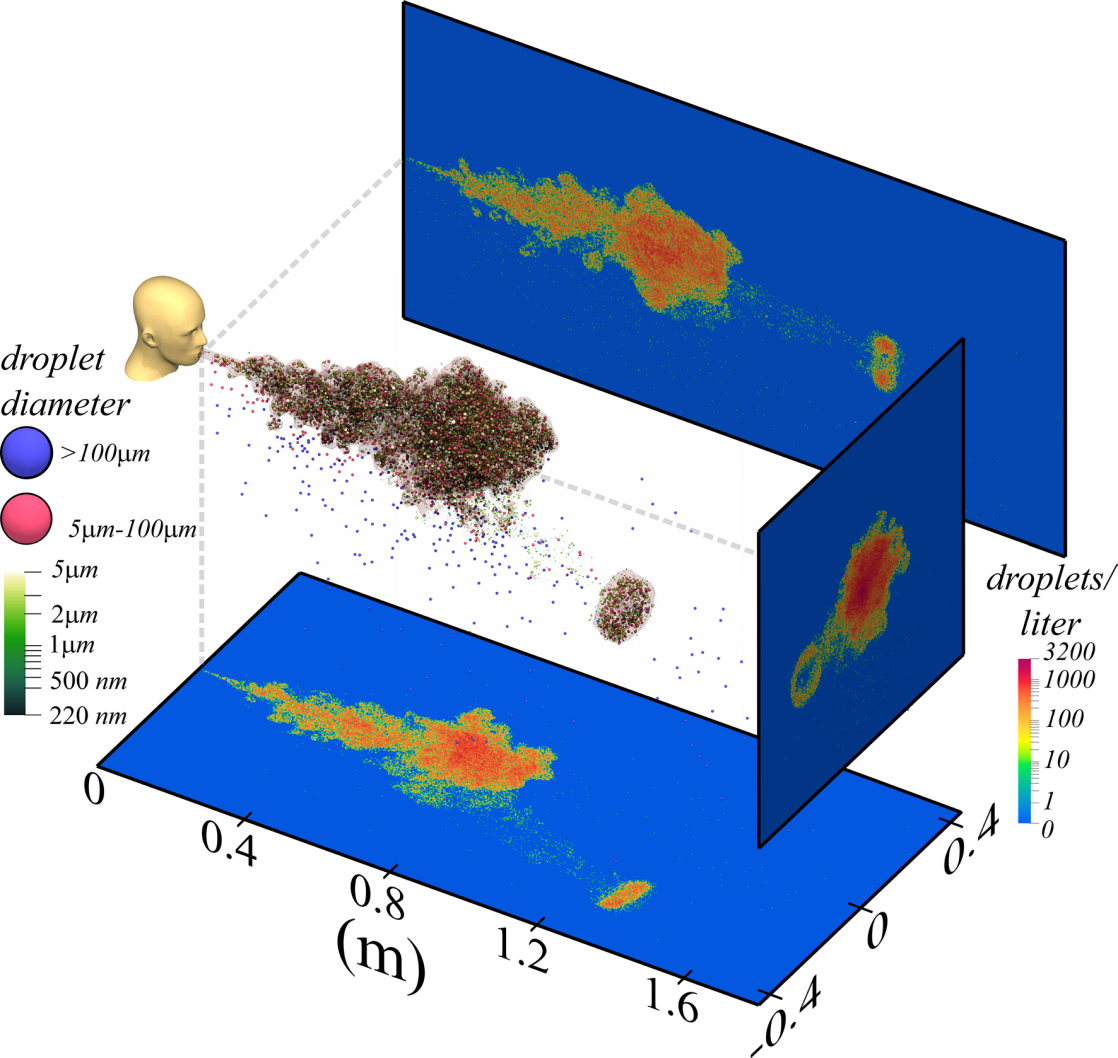
Droplets engulfed within the puff from a 1 l cough/sneeze 0.54 s after ejection. Droplets are colored by their size, with large droplets ($>5\,\upmu$m) given uniform size for clarity. The structure of the warm puff is shown by a temperature iso-surface of $20.15$ °C in light red. The three planes show projections of droplet number density (i.e., number of droplets per unit volume).

## Results

### Structure of the ejected droplet cloud

High-fidelity, large-eddy simulations of a cough or sneeze provide an unprecedented opportunity to study their turbulent evolution. Figure 1 shows a simulated cough/sneeze with an exhalation volume of 1 liter at an average velocity of 20 m s${^{-1}}$ containing about 61,650 droplets ranging between 1 and 1000 microns. A Pareto distribution $N_e(D) = B/D^2$ is used to determine the diameter of the ejected droplets^14^. With $B=0.061\,$m, the ejected droplet volume is $13.2 \, \,\upmu$L. While the ejected puff and droplets are at $35$°C, the ambient is at $20$°C. The structure of the puff, extracted using a temperature iso-surface of $20.15$°C, is clearly indicative of turbulent flow. Viewed at 0.54 s, the bulk of the puff remains coherent, except for a portion that separates as a vortex ring and travels at a faster speed towards the lower-right. Five other nearly identical coughs/sneezes (that differ only in the small random initial perturbation) were simulated, and the results display a diverse behavior. Some puffs exhibit a fast-moving detached portion heading in a different direction, while others remain coherent without a detached portion. Both behaviors have been observed experimentally^15–19^.

Also shown are ejected droplets colored according to their size. Larger droplets, colored blue ($D>100\,\upmu$m), overshoot the puff, reach farther distances, and quickly fall out. Smaller droplets, colored green ($D<5\,\upmu$m) and red ($5\,\upmu$m $<D<100\,\upmu$m), remain afloat and occupy the entire puff. Droplets that remain suspended within the fast-moving portion offer a mechanism by which droplets are transported to farther extents. The three number density projections show distinct variation, including the imprint of the peeled-off portion with its center devoid of droplets. This brings an important point that while droplets are well-distributed within the puff, without any bias or stratification, their distribution in any individual realization is not uniform. Droplets are observed to preferentially accumulate in strain-dominated regions as they are spun out by turbulent eddies and such de-mixing by turbulence is now well-understood^20–22^. Nevertheless, smaller droplets remain suspended for long times^17,23–29^ and henceforth will be referred to as the *droplet cloud*.

### Center and radius of the droplet cloud

**Figure 2:**
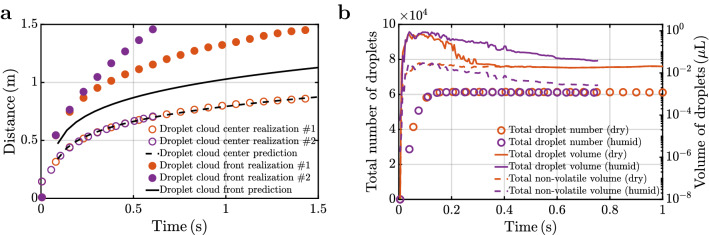
(**a**) Droplet cloud streamwise location of the center and farthest extent from realization #1 (orange circle, orange filled circle) and realization #2 (violet circle, violet filled circle). Corresponding theoretical predictions^14^ are shown as dashed and solid lines, respectively. (**b**) Total number (orange circle, violet circle), total volume (orange line, violet line), and non-volatile volume (orage dashed line, violet dashed line) of droplets within the droplet cloud for the (dry, humid) cases.

The streamwise center (open symbols) and maximum extent (filled symbols) of the droplet cloud are plotted in Fig. 2a for two different realizations (orange versus purple). While the center is in good agreement between all realizations, it is remarkable that the farthest extent shows substantial variation, and the difference continues to grow over time. We now evaluate our ability to predict the dynamics of the droplet cloud. According to the theory of^14,15^, the streamwise location of the center of the droplet cloud is given by

1 $$\begin{aligned} \frac{z_{\text {c}}(t) -z_{\text {inj}} + z_{\text {vo}}}{z_{\text {vo}}} = \left( \frac{t -t_{\text {inj}}+ t_{\text {vo}}}{t_{\text {vo}}} \right) ^\frac{1}{4+C} \, , \end{aligned}$$

where $z_{\text {c}}$ is the distance traveled by the droplet cloud. In the theory, ejection is instantaneous, whereas in reality and in simulations, ejection extends over a short period. In the simulations, $t_{\text {inj}}=0.085 \,$s represents the time when the bulk of the ejection is complete, and the corresponding distance covered is $z_{\text {inj}} = 0.33 \,$m. ${t}_{\text {vo}}=0.002 \,$ s and ${z}_{\text {vo}}=0.26\,$ m are empirical constants extracted from the simulations and represent the virtual origin time and location.

The theoretical estimate of the farthest extent of the droplet cloud can be obtained as the sum of the cloud center and radius as^14^

2 $$\begin{aligned} z_{\text {max}}(t) = z_{\text {c}} (t) + \alpha \, (z_{\text {c}}(t) -z_{\text {inj}} + z_{\text {vo}}) \, {.} \end{aligned}$$

The entrainment coefficient $\alpha = 0.24$ measures the rate at which the volume of the puff increases through entrainment of ambient fluid. The drag parameter $C = 0.22$ measures the frictional loss of momentum. Using these values obtained from the simulations, the predictions of equations (1) and (2) are plotted in Fig. 2a with dashed ($z_{\text {c}}$) and solid black lines ($z_{\text {max}}$). $z_{\text {max}}$ must be correctly interpreted as a lower bound. Due to the chaotic nature of the flow, the maximum droplet extent varies substantially across different realizations, but remains consistently greater than $z_{\text {max}}$. In contrast, the time evolution of the cloud center varies little and is well predicted by the theoretical power-law.

### Influence of humidity on droplet evaporation rate

In the simulation, the diameter *D* of an evaporating droplet is computed as^14,30–33^

3 $$\begin{aligned} \dfrac{\text {d} D}{\text {d} t} = - \, \frac{k'}{2 D} \quad \text {where} \quad k' = k'_{\text {st}} \dfrac{Nu}{Nu_{\text {st}}} \left( 1 - \psi _0 \frac{D^3_{0}}{D^3}\right) . \end{aligned}$$

The effective evaporation coefficient $k'$ is written as a product of three factors. The Stokes value $k'_{\text {st}}=4 {\mathcal {D}}_{\text {a}} {{Nu}}_{\text {st}} \ln ( 1+B_{\text {m}} ) / \uprho$ on its own is appropriate in the case of small droplet Reynolds number and in the absence of non-volatiles. ${\mathcal {D}}_{\text {a}}$ is the diffusion coefficient of water vapor, $\uprho$ is the water-to-air density ratio, and $B_m$ is the Spalding mass number. The ratio $Nu/Nu_{\text {st}}$ accounts for finite Reynolds number effects, where a simple heat transfer model $Nu = Nu_{\text {st}}(1+0.3 Re^{1/2} Pr^{1/3})$ is used. Here $Pr=0.72$ is the Prandtl number of air and *Re* is the droplet Reynolds number. As droplet diameter decreases due to evaporation, and as relative velocity decreases, *Re* becomes smaller than unity and $Nu/Nu_{\text {st}} \rightarrow 1$. The factor within the parenthesis accounts for the presence of non-volatiles. $\psi _0$ is the volume fraction of non-volatiles in the droplets at the time of ejection, which is taken to be $1\%$^34^, and $D_0$ is the initial droplet diameter whose current diameter is *D*. Immediately after ejection, the effect of non-volatiles is quite small and the droplet diameter closely follows the $D^2$-law: $D^2 = D_0^2 - k' \, t$. The evaporation rate decreases to zero as the droplet approaches its terminal diameter of $D_{\text {nv}} = D_0 \, \psi _0^{1/3}$. Upon near complete evaporation, the droplet is taken to become a *droplet nucleus*^28,35,36^.

Results from two different evaporative environments will be presented: $k'_{\text {st}} = 2.5 \times 10^{-7} \,$m$^2$s$^{-1}$ models a *dry* environment that promotes rapid evaporation and $k'_{\text {st}} = 1.0 \times 10^{-9} \,$m$^2$s$^{-1}$ models a *humid* environment. The total number of droplets within the cloud, shown as circles in Fig. 2b, after an initial increase remains nearly constant indicating that larger droplets have exited early. The total number of droplets is slightly lower in the humid case (purple circles) due to few more droplets, that remain large on account of slower evaporation, escaping the cloud. The droplet nuclei that form the cloud are sufficiently small that the flow and turbulence keep them afloat for a very long time.

**Figure 3:**
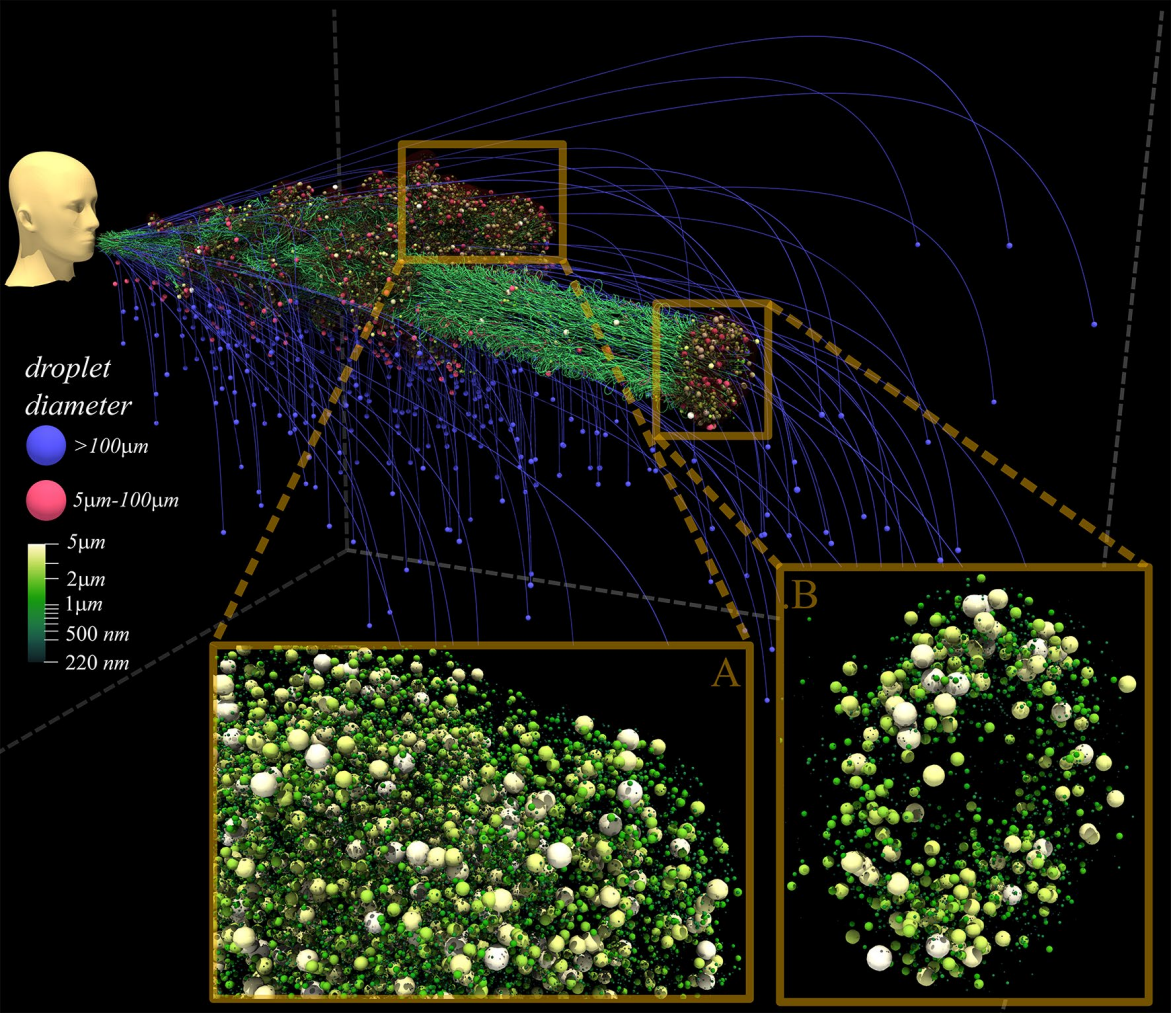
Same as Fig. 1 but with droplet trajectories colored according to droplet size. The trajectory of a large droplet (blue dot) is nearly unaffected by the puff and depends only on the initial droplet velocity. At the instant shown ($0.54\,$s), the velocity of large droplets is predominantly downward. Smaller droplets are suspended by the flow and undergo chaotic motion within the main body of the puff and follow spiraling trajectories for the separated, fast-moving vortex ring-like structure. The insets highlight the diameter range of airborne droplets at 1000*X* magnification. See movie S1 in supplementary information.

The droplet trajectories are shown in Fig. 3, where two distinct droplet behaviors are observed: (i) near-ballistic motion of large droplets ($D>100 \, \,\upmu$m) and (ii) chaotic motion of smaller droplets^27^. Even those large droplets that appear far ahead and at the same elevation as the mouth, are in fact falling rapidly. This supports the earlier assertion that the larger ballistic droplets will soon fall down and deposit on surfaces. The spiraling motion of droplets^27^, most visible for those transported by the fast-moving vortex ring, provide a clear mechanism by which turbulent eddies can keep the droplet nuclei afloat. Close-ups of the small droplets (${D}<5 \, \,\upmu$m), in both the main body and the vortex ring, are shown, where delicate features, such as the cork-screw-like motion of droplets behind the fast-moving portion, can be observed.

In both cases, the total volume of droplets reaches about the same peak and decays due to both evaporation and droplets leaving the cloud^37^. While the number of droplets within the cloud nearly accounts for all ejected droplets, the total volume is substantially lower than the ejected volume. This difference is largely due to droplets that are outside the cloud, though few in number, being much larger than those that remain within. The post-peak decay is more rapid under dry condition where all droplets within the cloud have become droplet nuclei, while evaporation is still proceeding in the humid condition.

Of particular importance is the volume of non-volatiles, which includes mucus, viruses, and solid particulates^34^, that remain airborne as a potential source of contagion. The total volume of airborne non-volatiles within the cloud is plotted in Fig. 2b for both dry (dashed orange line) and humid (dashed purple line) conditions. Under dry condition, the near-constant volume of non-volatiles, and its agreement with the total volume, confirms complete evaporation. It also indicates that the potentially infectious matter remains undiminished after the initial period. On the other hand, under humid condition, there is ongoing evaporation and droplet fall-out. We observe the important effect that the airborne non-volatile volume is smaller under humid condition due to increased droplet fall-out.

### Theoretical model

Theoretical analysis^14^ leads to the following two simple relations:

4 $$\begin{aligned} D_{\text {evap}}(t)= & {} \sqrt{\dfrac{k' (t-t_{\text {inj}}) \, \psi _0^{2/3}}{1 - \psi _0^{2/3}}} \, , \end{aligned}$$

5 $$\begin{aligned} D_{\text {exit}}(t)= & {} \sqrt{ \dfrac{A}{t-t_{\text {inj}}} \left( \dfrac{t-t_{\text {inj}}+t_{\text {vo}}}{t_{\text {vo}}}\right) ^{\frac{1}{4+C}} - \dfrac{k'}{2} (t-t_{\text {inj}}) }\, , \end{aligned}$$

$D_{\text {evap}}$ corresponds to a limiting diameter where all droplets of smaller size (i.e., $D \le D_{\text {evap}}$) can be considered to have fully evaporated. Similarly, all droplets larger than $D_{\text {exit}}$ can be considered to have fallen out. With increasing time, even larger droplets fully evaporate and therefore $D_{\text {evap}}$ increases with time. On the other hand, $D_{\text {exit}}$ decreases with time, since with the passage of time even smaller-sized droplets fall-out of the cloud. There exists a time $t_{\text {lim}}$ where $D_{\text {evap}}(t_{\text {lim}}) = D_{\text {exit}}(t_{\text {lim}}) = D_{\text {lim}}$. For short times ($t \le t_{\text {lim}}$), the cloud contains fully-evaporated droplet nuclei and droplets that are still evaporating:

6 $$\begin{aligned} t \le t_{\text {lim}}: \quad \underbrace{D \le D_{\text {evap}}}_{\text {nuclei}} \quad \underbrace{D_{\text {evap}} {<} D \le D_{\text {exit}}}_{\text {evaporating}} \quad \underbrace{D_{\text {exit}} {<} D}_{\text {exited}}. \end{aligned}$$

For times beyond $t_{\text {lim}}$, all droplets within the cloud have fully evaporated:

7 $$\begin{aligned} t \ge t_{\text {lim}}: \quad \underbrace{D \le D_{\text {lim}}}_{\text {nuclei}} \quad \underbrace{D_{\text {lim}} {<} D}_{\text {exited}} \, . \end{aligned}$$

In obtaining equation (4) the theory assumes: (i) droplets were ejected at the same instance, and (ii) $k'$ to be a constant. In reality, $k'$ is time-dependent and varies with *Re* and the increasing volume fraction of non-volatiles. Similarly, in obtaining equation (5) the theory assumes: (iii) the fall-out distance to be the radius of the cloud, (iv) droplets to be initially ejected horizontally, (v) the effect of non-volatiles to be negligible, and (vi) the effect of fluid velocity to be small. Therefore, we make the following two adjustments to the theory. First, in equations (4) and (5) we replace $k'$ by $k'_{\text {ef}} = 2.5 k'_{\text {st}}$. Note that this approximation does not affect the size of fully-evaporated droplet nuclei. The second adjustment pertains to the constant $A = 18 \delta \nu _{\text {a}} \alpha {z}_{\text {vo}}/(\uprho g)$, where $\nu _{\text {a}}$ is the kinematic viscosity of air and *g* the gravitational acceleration. To account for the neglected effects (iii) to (vi) a free parameter $\delta \approx 3$ is introduced, which is observed to yield good prediction.

### Importance of ambient humidity

Under dry condition, the theory yields $t_{\text {lim}} = 0.3 \,$s in good agreement with the simulation result presented in Fig. 2b. The corresponding $D_{\text {lim}} = 80.6 \, \,\upmu$m, whose original size at ejection is $D_{\text {0,lim}} = 373 \, \,\upmu$m. I.e, even droplets as large as 373 microns at ejection are still within the cloud. The theory predicts the total non-volatile volume to be $0.022 \, \,\upmu$L, which is in reasonable agreement with the computed results. However, the settling velocity of droplet nuclei, whose diameter is larger than $20 \, \,\upmu$m, exceeds $1 \,$cm s$^{-1}$^14^ and thus may continue to slowly fall-out of the cloud over minutes, in the absence of strong turbulence.

Under humid condition, the theory yields $t_{\text {lim}} =5.0 \,$s and $D_{\text {lim}} = 24.1 \, \,\upmu$m. Thus, droplet nuclei between $24.1 \, \,\upmu$m and $80.6 \, \,\upmu$m, which remain airborne under dry condition are lost - a theoretical prediction that is again consistent with the simulation results. A more direct evaluation of the theory is obtained for $t = 0.54 \,$s. From (4) and (5) we obtain $D_{\text {evap}} = 7.65 \, \,\upmu$m and $D_{\text {exit}} = 192.7 \, \,\upmu$m. I.e., only droplets smaller than $7.65 \, \,\upmu$m have fully evaporated. However, the upper limit $D_{\text {exit}}$ is surprisingly larger than $80.6 \, \,\upmu$m, which is the upper limit under dry condition. This puzzlement can be resolved by observing that $D_{\text {exit}} = 192.7 \, \,\upmu$m corresponds to only a slightly larger ejected diameter of $D_{\text {0,exit}} = 195.8 \, \,\upmu$m. I.e., the droplets have evaporated little under humid condition, in contrast to dry condition where even a $D_{\text {0,lim}} = 373 \, \,\upmu$m droplet has fully evaporated. Under humid condition, the theory predicts a non-volatile volume of $0.006 \, \,\upmu$L and ongoing evaporation at $t = 0.54 \,$s. Both predictions are in agreement with the simulation.

The volume of non-volatiles that remain airborne is about 4 times lower under humid condition. In the presence of ambient currents^38,39^, airborne droplet nuclei can travel farther than two meters. Thus, rapid initial evaporation under dry conditions can leave a larger volume of potentially infectious matter to be carried around.

### Theoretical prediction

**Figure 4:**
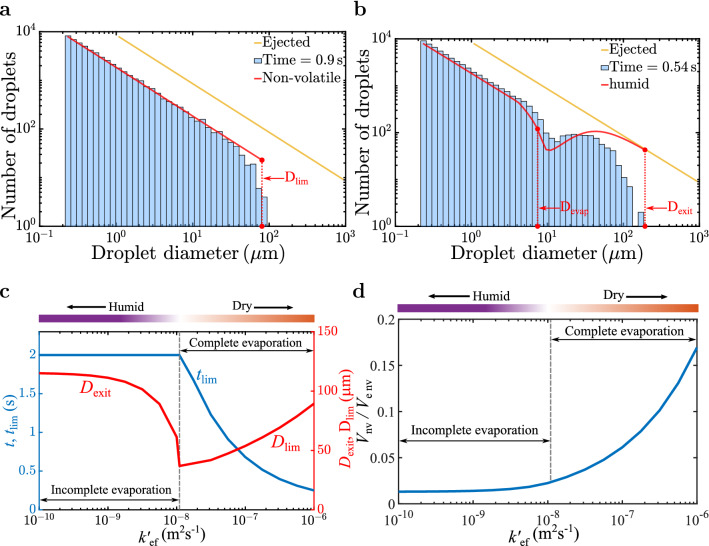
Number of droplets versus droplet diameter *D* for the (**a**) dry and (**b**) humid cases. The yellow line corresponds to the initial ejected size distribution. (**a**) Distribution at $t=0.9\,$s matches well with the theory^14^ (red line). $D_{\text {lim}}$ is the largest droplet diameter within the cloud. (**b**) Distribution at $t=0.54\,$s against the theoretical prediction (red line). (**c**) $t_{lim}$ (when $t<2$s) for varying values of $k'_{\text {ef}}$. Also shown are $D_{\text {exit}}$ and $D_{\text {lim}}$ vs. $k'_{\text {ef}}$. (**d**) Ratio of non-volatile volume ($V_{\text {nv}}$) to ejected non-volatile volume ($V_{\text {e\,nv}}$) vs. $k'_{\text {ef}}$ as predicted by the theory at $t = 2 \,$ s.

The number of droplets spectra within the cloud as a function of droplet size is shown in Fig. 4 where the *y*-axis corresponds to the number of droplets within a droplet diameter bin of $[D/2 - 2D]$. The initial Pareto distribution appears as a yellow line. Under dry condition (frame a), the simulation results shown as the histogram are in good agreement with the theoretical prediction (red line)^14^. For $t > 0.3 \,$s all airborne droplets are fully evaporated and the spectra is simply the original distribution left-shifted to the new diameter. The largest computed droplet nucleus diameter is in good agreement with the theoretical prediction of $D_{\text {lim}} = 80.6 \, \,\upmu$m.

For the humid condition (frame b), $t = 0.54 \, \text {s} < t_{\text {lim}}$, and therefore two distinct regimes are seen, in accordance with equation (6). The spectra of fully-evaporated droplet nuclei appears as a shifted straight line and extends up to $D_{\text {evap}} = 7.65 \, \,\upmu$m in good agreement with the theory. In the regime where droplets are still evaporating, agreement is reasonable with theory and simulation displaying a characteristic dip. However, the theoretical dip is larger, due to the assumption $k'_{\text {ef}} = 2.5 k'_{\text {st}}$. A better model would be to vary the factor 2.5 as a function of droplet diameter. The theoretical spectra approaches the ejection spectra before dropping to zero at $D_{\text {exit}} = 192.7 \, \,\upmu$m. While the predicted maximum droplet diameter is in good agreement with the computed value, the theoretical spectra does not show the faster decay observed in the histogram. This difference is due to the assumption that small droplets of $D < D_{\text {exit}}$ remain entirely within the cloud, which is not accurate, since a fraction of these droplets would have also fallen out.

Under dry condition, before reaching the terminal state, the spectra qualitatively resembles that shown in frame b. At early time, a dip starts at the left end of the spectra. With increasing time, the location of the dip travels right closely following $D_{\text {evap}}(t)$ and finally at $t=t_{\text {lim}}$, when $D_{\text {evap}} = D_{\text {exit}} = D_{\text {lim}}$, the spectra reaches the terminal state shown in Fig. 4a. The spectra from all realizations follow this evolution qualitatively (see supplementary movies S2 and S3).

In essence, irrespective of ambient conditions, the number spectra of airborne droplet nuclei will reach the terminal state shortly after ejection. In log-log scale, the terminal spectra is simply the left-shifted initial spectra, where the left-shift depends only on the fraction of non-volatiles in the initial ejection. The upper diameter limit of the terminal spectra is given by $D_{\text {lim}}$, which primarily depends on $k'_{\text {ef}}$. For the prediction of long time airborne droplet nuclei, only the terminal spectra is of interest, which is well predicted by theory^14^. Instead of the Pareto distribution, if the ejected droplets had followed a log-normal or a different distribution, the corresponding terminal spectra will still be a left-shifted spectra with an upper diameter limit of $D_{\text {lim}}$.

## Discussion

We conclude with the following remarks: (i) While humid ambient conditions can extend the life of droplets^27^, they result in a smaller non-volatile volume due to enhanced settling. We observe dry ambient conditions to increase by more than four times the number of airborne potentially virus-laden nuclei, as a result of reduced droplet fall-out through rapid evaporation. (ii) Even under nearly identical conditions, coughs/sneezes can vary substantially due to their chaotic nature. The ejected droplets self-sort themselves into larger droplets that quickly fall-out and smaller droplets that fully-evaporate to become droplet nuclei. Smaller droplet nuclei form a cloud that remains airborne for a long time and travels long distances^17,23–27^. Occasionally, droplets are carried to greater distances by fast-moving, vortex ring-like break-away portions of the puff. Global parameters, such as the center of the droplet cloud, vary little across realizations and are well-predicted by theory^14^. (iii) The theory of^14^ provides a valuable tool for accurately predicting the number of droplet nuclei that remain airborne for a long time. The theory offers three simple predictions: first, the number density spectrum of airborne droplet nuclei is simply the left-shifted original ejected droplet spectrum, Second, all the airborne droplet nuclei have reached their terminal fully-evaporated state, and third, droplet nuclei of size larger than $D_{exit}$ have fallen out of the cloud. These predictions can be used to quickly obtain a good estimate of the volume of long-term airborne droplet nuclei and the viral content within them under various scenarios without the need for full fledged experiments or simulations.

For example, the theory can now be used to calculate the fate of ejected droplets, say $2 \,$s after ejection, for a range of $k'_{\text {ef}}$. Figure 4c shows a plot of the minimum between $t= 2 \,$s and $t_{\text {lim}}$. For $k'_{\text {ef}} > 1.1 \times 10^{-8}\,$m$^2$s$^{-1}$, $t_{\text {lim}}$ is smaller than $2 \,$s, while for smaller $k'_{\text {ef}}$, evaporation is not yet complete and $t_{\text {lim}}>2 \,$s. Also plotted are $D_{\text {exit}}$ for $k'_{\text {ef}} < 1.1 \times 10^{-8}\,$m$^2$s$^{-1}$ and $D_{\text {lim}}$ for $k'_{\text {ef}} > 1.1 \times 10^{-8}\,$m$^2$s$^{-1}$. With increasing $k'_{\text {ef}}$ we observe $D_{\text {exit}}$ to decrease since droplets avoid fall-out due to rapid evaporation. Whereas, $D_{\text {lim}}$ increases, since the fall-out rate decreases and increasingly larger droplets remain airborne. From the above, the volume of non-volatiles that remain airborne, normalized by the ejected non-volatile volume, is calculated and plotted in Fig. 4d. A monotonic increase in airborne non-volatile volume with increasing $k'_{\text {ef}}$ is seen. For $k'_{\text {ef}} = 10^{-6}\,$m$^2$s$^{-1}$ about 17% of ejected viral content still remains airborne, but under humid condition this fraction decreases by a factor of about 4.

The theory can also be used to evaluate the location and size of the droplet could. For example, by $t = 2 \,$s the center of the droplet cloud would be at a distance of $1.0 \,$m and its average radius would be $0.26 \,$m. However, the farthest extent of the cloud can be expected to exceed $1.75 \,$m, or even more, in some realizations. Other ejection scenarios can be considered as well. For example, if we consider a more intense cough/sneeze of volume 3 liters with an average velocity of 30 m s$^{-1}$, the model predicts the center to have reached $1.72 \,$m after $2 \,$s with the radius of the cloud being $0.4 \,$m. Again, the farthest extent of the airborne droplets will be much larger than $2.12 \,$m. The aforementioned ejection volume of 3 liters may be considered to be an average ejection volume among adults^40^. Based on the current theoretical framework, we now provide three levels for risk of airborne contagion based on the separation distance between individuals and for an average ejected volume of 3 liters. The three levels are high risk, medium risk, and low risk.

A high risk of contagion occurs when the separation distance is less than the distance traveled by the front of the coherent (non-separated) droplet cloud. For an average cough/sneeze the theory^14^ predicts this distance to be about $2.2 \,$m, however, the simulations indicate that the actual distance may be much larger (see Fig. 2a). A better estimate would put this distance at about $2.8 \,$m. A medium risk of contagion occurs when the separation distance is less than that traveled by the potentially detached droplet cloud. Based on our simulations, this distance can be up to 120% to 130% of the maximum distance traveled by the front of the coherent droplet cloud, which amounts to about 3.4 to $3.6 \,$m. Finally, a low risk of contagion would correspond to separation distances in excess of $3.6 \,$m.

The implications for social distancing guidelines could be substantial, as the classical recommendation of 2m may need to be re-evaluated in light of the chaotic nature of puff dynamics. Probabilistic outlier-events do occur and may need to be taken into account when assessing the risk of contagion^41^. The high sensitivity to perturbations in the exhalation process would be reflected as an increased standard deviation in the probabilistic infection risk models.

Most importantly, the present results also highlight the important role the environment plays, especially through the evaporation process. Since the viruses within the droplets do not disappear with evaporation but remain airborne for a very long time, rapid evaporation greatly reduces droplet fall-out. As a result, under otherwise identical conditions, dry weather promotes spreading of up to four times more airborne virus-laden droplets than humid weather. This provides an explanation to the increased rate of airborne transmission during the winter months, when people spend more time indoors with drier ambient conditions^42,43^.

## Methods

We use the spectral element Nek5000 code^44^ to solve the equations of mass, momentum, and energy conservation for the puff. The droplets are tracked using the Euler-Lagrange point-particle approach in a domain of size $1.8\times 1.8\times 1.8$ (m$^3$) resolved by $120\times 120\times 960$ grid points along the vertical, cross-stream, and flow directions, respectively. The ejection velocity profile during a cough/sneeze varies with time and is adopted from the experiments of^45^. The large injection Reynolds number $Re_{\text {inj}}=45000$, based on a peak ejection velocity of 30.4 m s$^{-1}$ and on a mouth diameter of 2.26 cm favors an LES approach with sub-grid modeling to account for the unresolved scales. Here, the dynamic Smagorinsky model is employed^46–48^. Droplets are modeled as spheres with a 1% non-volatile content at ejection. The diameter of the evaporating droplet is followed by numerically integrating equation (3). A total of 61650 droplets ranging from 1 to 1000 microns are ejected over a period of 0.2 s. Within each time step, the volume of ejected droplets is maintained proportional to the volume of ejected fluid, so that the droplet volume fraction remains a constant at $1.3 \times 10^{-5}$. To model the effect of unresolved scales on droplets, we implement the Langevin model^49,50^ through stochastic, sub-grid scale velocity perturbations.

## Supplementary Information

Supplementary Video S1.

Supplementary Video S2.

Supplementary Video S3.

## Data Availability

The simulation data that support the findings of this study will be made available in Open Science Framework osf.io. Source codes are available at https://github.com/Nek5000/Nek5000 and at https://github.com/josalinas/ppiclF.
